# Effect of the Xpert MTB/RIF on the detection of pulmonary tuberculosis cases and rifampicin resistance in Shanghai, China

**DOI:** 10.1186/s12879-020-4871-9

**Published:** 2020-02-18

**Authors:** Zheyuan Wu, Zulma Vanessa Rueda, Tao Li, Zurong Zhang, Yuan Jiang, Wei Sha, Fangyou Yu, Jing Chen, Qichao Pan, Xin Shen, Zheng’an Yuan

**Affiliations:** 1 Department of Tuberculosis Control, Shanghai Municiple Center for Disease Control and Prevention, Shanghai, China; 2 Universidad Pontificia Bolivariana, Medellín, Colombia; 3 Chinese Center for Disease Control and Prevention, Beijing, China; 4 Laboratory of Tuberculosis Diagnosis, Shanghai Municiple Center for Disease Control and Prevention, Shanghai, China; 5 Shanghai Key Laboratory of Tuberculosis, Clinic and Research Center of Tuberculosis, Shanghai Pulmonary Hospital, Tongji University School of Medicine, Shanghai, China; 6 Department of Clinical Laboratory Medicine, Shanghai Pulmonary Hospital, Tongji University School of Medicine, Shanghai, China; 7 Shanghai Municiple Center for Disease Control and Prevention, Shanghai, China

**Keywords:** SORT IT, Xpert MTB/RIF, TB, Delay, Rifampicin resistant, Tuberculosis

## Abstract

**Background:**

Xpert MTB/RIF (Xpert) is an automated molecular test recommended by World Health Organization (WHO) for diagnosis of tuberculosis (TB). This study evaluated the effect of Xpert implementation on the detection of pulmonary TB (PTB) and rifampicin-resistant TB (RR-TB) cases in Shanghai, China.

**Methods:**

Xpert was routinely implemented in 2018 for all presumptive PTB patients. All PTB patients above 15 years-old identified within the Provincial TB Control Program during the first half of each of 2017 and 2018, were enrolled to compare the difference in proportions of bacteriological confirmation, patients with drug susceptibility test (DST) results for rifampicin (ie, DST coverage) and RR-TB detection before and after Xpert’s implementation.

**Results:**

A total of 6047 PTB patients were included in the analysis with 1691 tested by Xpert in 2018. Percentages of bacteriological confirmation, DST coverage and RR-TB detection in 2017 and 2018 were 50% vs. 59%, 36% vs. 49% and 2% vs. 3%, respectively (all *p*-values < 0.05). Among 1103 PTB patients who completed sputum smear, culture and Xpert testing in 2018, Xpert detected an additional 121 (11%) PTB patients who were negative by smear and culture, but missed 248 (23%) smear and/or culture positive patients. Besides, it accounted for an increase of 9% in DST coverage and 1% in RR-TB detection. The median time from first visit to a TB hospital to RR-TB detection was 62 days (interquartile range -IQR 48–84.2) in 2017 vs. 9 days (IQR 2–45.7) in 2018 (p-value < 0.001). In the multivariate model, using Xpert was associated with decreased time to RR-TB detection (adjusted hazard ratio = 4.62, 95% confidence interval: 3.18–6.71).

**Conclusions:**

Integrating Xpert with smear, culture and culture-based DST in a routine setting significantly increased bacteriological confirmation, DST coverage and RR-TB detection with a dramatic reduction in the time to RR-TB diagnosis in Shanghai, China. Our findings can be useful for other regions that attempt to integrate Xpert into routine PTB and RR-TB case-finding cascade. Further study should focus on the identification and elimination of operational level challenges to fully utilize the benefit of rapid diagnosis by Xpert.

## Background

China has the second highest tuberculosis (TB) and multidrug-resistant/rifampicin-resistant TB (MDR/RR-TB) incidence in the world [1]. Early diagnosis and effective treatment are crucial to stop transmission and to succeed with the End TB strategy [2]. Conventionally, sputum smear microscopy, culture and culture-based drug susceptibility test (DST) are used to diagnose TB and MDR/RR-TB. However, smear microscopy has low sensitivity and specificity compared to culture, culture usually takes weeks to report results, and DST results can take even longer [3].

Xpert® MTB/RIF assay (Cepheid, USA) (Xpert) is a rapid test for TB diagnosis currently endorsed by the WHO [4], which simultaneously tests for *Mycobacterium tuberculosis* (MTB) and resistance to rifampicin (RIF), providing results within 2 h. Clinical validation trials have demonstrated high sensitivity and specificity of the test for both MTB and RIF-resistance detection in different settings [4–7]. Several studies have even suggested that Xpert outperforms culture in salivary sputum [8, 9] and can detect low-levels of RIF-resistance, which might be missed by culture-based DST [10, 11]. The WHO has recommended that Xpert be the initial test in adults and children with presumptive MDR-TB or HIV-associated TB [4]. The use of Xpert in decentralized settings has significantly increased proportions of bacteriologically confirmed TB and RR-TB and reduced the time to treatment initiation [12–15].

In Shanghai, China, sputum specimens are collected for all presumptive PTB patients to perform smear microscopy and culture. Culture-based drug susceptibility test (DST) for isoniazid (H), rifampicin (R), ethambutol (E), and streptomycin (S) is routinely conducted for culture positive MTB strains to identify MDR-TB [16, 17]. Without any use of the WHO recommended rapid diagnostic methods, the percentage of bacteriologically confirmed PTB patients and DST coverage has been reported at approximately 40% [18]. Additionally, the median time required to complete culture-based DST is 41 days, which accounts for 23% of time from TB diagnosis to MDR-TB treatment [16]. Lastly, delayed case identification and the initiation of appropriate treatment may lead to a higher risk of recent transmission and poor TB control [19].

In 2018, Shanghai launched a large scale project funded by the local government to test all presumptive PTB patients using Xpert at their first visit to designated TB hospitals. It aimed to increase bacteriological confirmation and DST coverage among TB patients and to shorten the delay of RR-TB detection. We conducted this study to evaluate the effect of Xpert implementation on the detection of PTB cases and RIF-resistance, including time to RR-TB detection and risk factors associated with RR-TB delay.

## Methods

### Study design

Primarily, this was a cross-sectional study utilizing routinely collected programmatic data for secondary analysis. In addition, we performed a retrospective cohort (time to event analysis) to identify time to RR-TB detection and the factors associated with it.

### General setting study population

There are 30 designated TB hospitals in Shanghai (four referral hospitals and 26 district hospitals) in charge of TB diagnosis and treatment, and more than 240 community health centers in charge of patient management (ie. DOTS). The Xpert project (detailed below) was initiated on January 1st, 2018. By November 2018 when the analysis was performed, only data of the first half of 2018 was available. Therefore, all PTB patients 15 years of age and above identified within the Provincial TB Control Program in the first half of 2018 were included in this study. Patients identified in the first half of 2017 were also enrolled for comparison.

### The provincial TB control program

All presumptive PTB patients (ie, cough for more than 2 weeks) detected in a general hospital or a community health center are referred to the district TB hospital where sputum smear, liquid culture, chest radiograph and other necessary tests are carried out for diagnosis. Culture positive strains are transferred to the TB reference laboratory at Shanghai Center for Disease Control and Prevention (CDC) to conduct a Lowenstein-Jensen culture for species identification and DST for first-line drugs (H/R/E/S); while one referral TB hospital, Shanghai Pulmonary Hospital, conducts liquid culture for species identification and DST for first-line (H/R/E/S) and 3 sec-line drugs (Aminoglycoside/Ofloxacin/Cycloserine). The TB reference laboratory at Shanghai CDC is responsible for the quality control of all TB laboratories in the 30 surrounding TB hospitals.

According to the national standard of diagnosis for PTB [20], confirmed PTB cases include individuals who are sputum smear microscopy positive, culture positive, molecular test positive or individuals who have pulmonary lesions of tuberculosis that have been confirmed by pathological examination (lung biopsy). Those who fail to meet criteria for confirmed PTB are clinically diagnosed if other pulmonary diseases are excluded or their chest radiograph supports active PTB and they have any of the below: 1. PTB signs like cough, expectoration, hemoptysis; 2. Immunology evidence like strong purified protein derivative (PPD) skin test reaction, positive Interferon-gamma release assay (IGRA), positive MTB anti-body test; or 3. extra-pulmonary TB confirmed by pathological examination.

RR-TB patients are subject to an expert panel [21] for final diagnosis and regimen design. All patients are treated under the supervision of doctors from the local community health center.

### The Xpert project

A total of 17 TB hospitals were equipped with Xpert platforms. Laboratory staff were trained by the reference laboratory of Shanghai CDC on the operation and reporting. Since January 1st, 2018, an Xpert test has been performed for all presumptive PTB patients along with sputum smear and culture at their first visit to one of these TB hospitals. Patients in hospitals without Xpert platforms were transferred to a nearby hospital where Xpert testing was available.

The Global Laboratory Initiative (GLI) and the WHO [3, 22] suggested that patients other than those at high risk of RR-TB (ie, previously treated patients; non-converters [smear positive at end of intensive phase]; and MDR-TB contacts) require a second Xpert test if the initial test was positive for RIF-resistance to control for potential errors. Therefore, new PTB patients in this study were at low risk of RR-TB and those with a RIF-resistant result were offered a second Xpert test to reduce false positive cases.

### Data collection and analysis

Data was extracted from the National TB management information system into Microsoft Excel® (Redmond, Washington, USA). A bacteriologically confirmed case was defined as a PTB case with positive results of smear, culture or Xpert. A DST covered case was defined as a PTB case with DST results for RIF. The delay of RR-TB detection was defined as time (days) from the date of first visit to a TB hospital to the date RIF-resistance was detected by either conventional DST or Xpert.

Analysis was performed using R® software (3.5.1). Demographic, clinical and diagnosis characteristics were analyzed and presented in frequencies and medians with an interquartile range (IQR) for categorical and continuous variables, respectively. Overall bacteriological confirmation and RR-TB detection was reported in frequencies. Comparison of frequencies was done using chi-square test, and medians using Wilcoxon rank-sum test. We estimated the probability of RR-TB detection using the Kaplan-Meier method and subgroups were compared using the log rank test. Cox proportional hazard model was used to investigate the factors associated with RR-TB detection. Unadjusted hazard ratios (HR) were calculated from a bivariate analysis. Adjusted hazard ratios were estimated by multivariate analysis. The final model was adjusted for all the variables from the bivariate analysis. A *p*-value of less than 0.05 was considered significant.

## Results

### Epidemiological and laboratory characteristics

There were 6357 PTB patients registered in the first half of each of 2017 and 2018. After excluding patients under 15 years old (31 cases) and those diagnosed as nontuberculous mycobacteria (NTM, 279 cases), 6047 PTB patients were included in the analysis (2017: 2938; 2018: 3109), with a median age of 44 years (IQR 27–62). The majority were male (68%), new patients (93%), local residents (54%) and registered in referral hospitals (69%), with no significant difference between 2017 and 2018.

The proportion of bacteriological confirmation and DST coverage in 2017 and 2018 were 50% vs. 59% and 36% vs. 49%, respectively. The RR-TB detection in the 2 years was 2 and 3% among new patients; and 6 and 11% among previously treated patients. There were 156 RR-TB patients detected, among them 119 (76%) were new cases. Proportions of bacteriological confirmation, DST coverage and RR-TB detection were significantly higher in 2018 than in 2017 (*p*-value < 0.05) (Table 1).

**Table 1:** Epidemiological characteristics of pulmonary tuberculosis patients notified in Shanghai, China, in the first half of each of 2017 and 2018*

Characteristics	Total n (%) (*n* = 6047)	PTB patients of 2017 n (%) (*n* = 2938)	PTB patients of 2018 n (%) (*n* = 3109)	*p*-value
Sex				0.912
Female	1910 (31.6)	930 (31.7)	980 (31.5)	
Male	4137 (68.4)	2008 (68.3)	2129 (68.5)	
Age				0.198
15–29	1887 (31.2)	904 (30.8)	983 (31.6)	
30–44	1190 (19.7)	578 (19.7)	612 (19.7)	
45–59	1171 (19.4)	601 (20.5)	570 (18.3)	
> =60	1799 (29.8)	855 (29.1)	944 (30.4)	
Hospital				0.928
County-level	1901 (31.4)	922 (31.4)	979 (31.5)	
Referral	4146 (68.6)	2016 (68.6)	2130 (68.5)	
Local resident				0.829
No	2781 (46.0)	1347 (45.8)	1434 (46.1)	
Yes	3266 (54.0)	1591 (54.2)	1675 (53.9)	
TB case				0.208
New	5608 (92.7)	2712 (92.3)	2896 (93.1)	
Previously treated	439 (7.3)	226 (7.7)	213 (6.9)	
Bacteriological confirmation				< 0.001
Yes	3290 (54.4)	1461 (49.7)	1829 (58.8)	
DST coverage				< 0.001
Yes	2573 (42.6)	1066 (36.3)	1507 (48.5)	
RR-TB detection				0.004
Yes	156 (2.6)	58 (2.0)	98 (3.2)	

*PTB* pulmonary tuberculosis

*Xpert* Xpert MTB/RIF

*MTB* Mycobacteria of tuberculosis

*RIF* Rifampicin

*RR-TB* Rifampicin-resistant tuberculosis

*DST* Drug susceptibility test

*Xpert was implemented in 2018

### Effect of Xpert on bacteriological confirmation, DST coverage and RR-TB detection in the first half of 2018

**Fig. 1:**
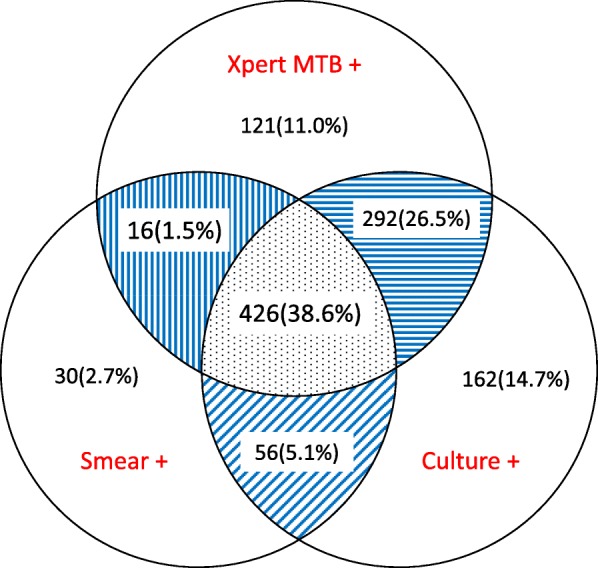
Agreement of sputum smear, culture and Xpert MTB in 1103 pulmonary tuberculosis patients with all tests in Shanghai, China, in the first half of 2018. Smear +: smear positive. Culture +: culture positive. Xpert MTB +: Xpert MTB positive. The blue lines represent the agreement between two diagnostic techniques used, and the dot area shows the agreement among all three tests

Among 1691 (54%) PTB patients who underwent for Xpert in 2018, 909 (54%) were MTB positive, and 69 (8%) RIF-resistant (Table 2). A total of 1103 PTB patients completed sputum smear, culture and Xpert tests. Fig. 1 shows that Xpert failed to detect 248 (23%) sputum smear and/or culture positive patients, but detected 121 (11%) PTB patients who were negative by smear and culture. In addition, Xpert detected 426 out of 482 (88%) smear-positive, culture-positive patients and 292 out of 454 (64%) smear-negative, culture-positive patients.

**Table 2:** Laboratory test results of pulmonary tuberculosis patients notified in Shanghai, China, in the first half of each of 2017 and 2018

Characteristics	Total n (%) (*n* = 6047)	PTB patients of 2017 n (%) (*n* = 2938)	PTB patients of 2018 n (%) (*n* = 3109)	*p*-value
Sputum smear				< 0.001
Negative	3784 (62.6)	1812 (61.7)	1972 (63.4)	
Positive	1802 (29.8)	850 (28.9)	952 (30.6)	
Not available	461 (7.6)	276 (9.4)	185 (6.0)	
Sputum culture				< 0.001
Negative	2123 (35.1)	1000 (34.0)	1123 (36.1)	
Positive	2786 (46.1)	1285 (43.7)	1501 (48.3)	
Not available	1138 (18.8)	653 (22.3)	485 (15.6)	
Xpert result for MTB				–
Negative	782 (12.9)	–	782 (25.2)	
Positive	909 (15.0)	–	909 (29.2)	
Not available	4356 (72.0)	2938 (100)	1418 (45.6)	
Xpert result for RIF resistance^a^				–
Susceptible	840 (92.4)	–	840 (92.4)	
Resistant	69 (7.6)	–	69 (7.6)	
Culture-based DST result for RIF resistance^b^				0.784
Susceptible	2188 (78.5)	1008 (78.4)	1180 (78.6)	
Resistant	118 (4.2)	58 (4.5)	60 (4.0)	
Not available	480 (17.2)	219 (17.0)	261 (17.4)	

*PTB* Pulmonary tuberculosis

*Xpert* Xpert MTB/RIF

*MTB* Mycobacteria of tuberculosis

*RIF* Rifampicin

*DST* Drug susceptibility test

^a^calculated among Xpert MTB positive patients

^b^calculated among culture positive patients

**Fig. 2:**
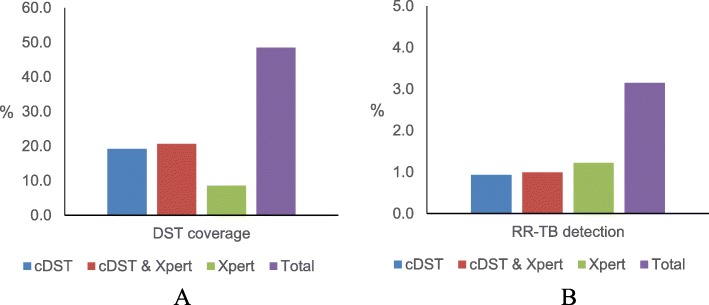
Drug susceptibility test coverage (A) and rifampicin-resistant tuberculosis detection (B) among pulmonary tuberculosis patients in Shanghai, China, in the first half of 2018. RR-TB = rifampicin-resistant tuberculosis. cDST = conventional drug susceptibility test. Xpert = Xpert MTB/RIF. Blue, green and red bars indicates the contribution of cDST alone, Xpert alone, and when both cDST & Xpert were done

Regarding DST coverage and RR-TB detection, Xpert accounted for an increase of 9 and 1%, respectively (Fig. 2). Among 52 new patients with RIF-resistant results from Xpert, 16 (31%) were verified with the second Xpert test: 15 remained RIF-resistant; 1 turned to RIF-sensitive but culture-based DST showed RIF-resistant. Of 36 without second Xpert test, 13 were RIF-resistant, 7 RIF-sensitive according to culture-based DST and the others had no further verification.

### Effect of Xpert on delay of RR-TB detection

**Fig. 3:**
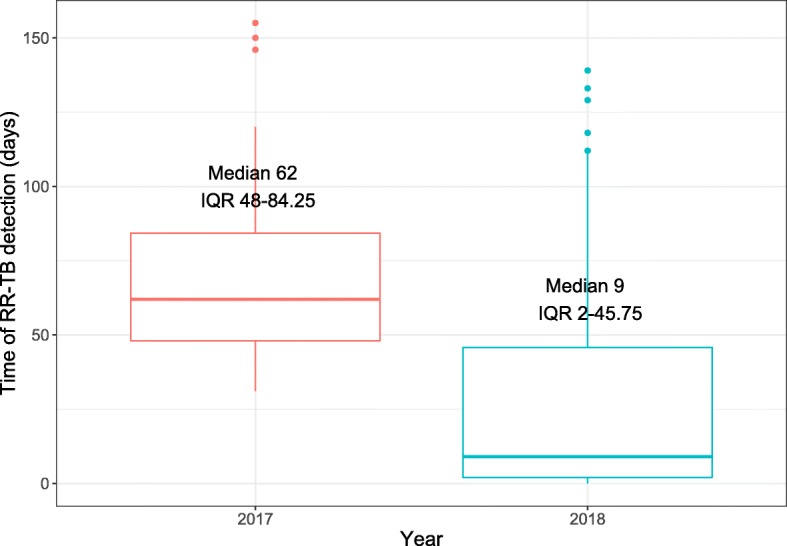
Time of rifampicin-resistant pulmonary tuberculosis detection in Shanghai, China, in the first half of each of 2017 and 2018*. Time of rifampicin-resistant pulmonary tuberculosis detection: days from the first visit to tuberculosis clinics to rifampicin-resistance was reported. RR-TB = rifampicin-resistant tuberculosis. * Xpert MTB/RIF was implemented in 2018

**Fig. 4:**
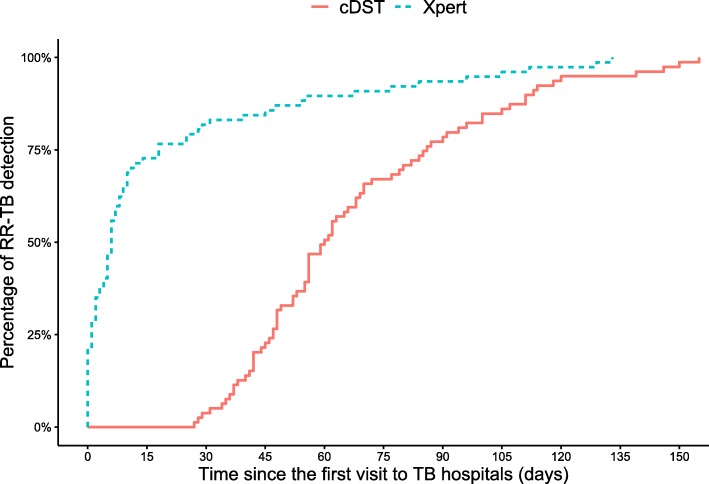
Kaplan-Meier for probability of rifampicin-resistant pulmonary tuberculosis detection since the first visit to TB hospitals using Xpert (*n* = 77, blue dashed line) and conventional drug susceptibility test (*n* = 79, red solid line). RR-TB = rifampicin-resistant tuberculosis. cDST = conventional drug susceptibility test. Xpert = Xpert MTB/RIF

The median of delay to RR-TB detection was 62 days (IQR 48–84) in 2017 vs. 9 days (IQR 2–45) in 2018 (Fig. 3, *p*-value < 0.001, Wilcoxon rank-sum test). When Xpert was used, the delay to RR-TB detection was significantly shorter compared to conventional DST (Fig. 4, *p*-value< 0.001, log-rank test).

In the multivariate model, utilization of Xpert was associated with decreased delay to RR-TB detection after adjustment for sex, age and other factors shown in the model (adjusted HR = 4.62, 95% CI 3.18–6.71) (Table 3). In addition, referral hospital and recurrent TB were also associated with decreased delay to RR-TB detection.

**Table 3:** Univariate and multivariate analysis of factors associated with RR-TB detection in Shanghai, China, in the first half of each of 2017 and 2018

Variables	Number of RR-TB patients (*n* = 156)	Unadjusted HR (95%CI)	Adjusted HR (95%CI)^a^
Sex			
Female	44	1.00	1.00
Male	112	1.05 (0.73–1.51)	0.88 (0.59–1.31)
Age			
15–29	48	1.00	1.00
30–44	36	0.97 (0.63–1.51)	1.37 (0.86–2.19)
45–59	45	0.98 (0.65–1.47)	0.93 (0.60–1.46)
> =60	27	0.75 (0.46–1.22)	1.08 (0.64–1.84)
Hospital			
County-level	47	1.00	1.00
Referral	109	2.65 (1.75–4.03)	2.92 (1.85–4.59)
Local resident			
No	86	1.00	1.00
Yes	70	0.97 (0.71–1.34)	1.13 (0.78–1.63)
TB case			
New	119	1.00	1.00
Recurrent	37	1.39 (0.28–1.19)	1.66 (1.11–2.50)
Sputum culture			
Not done	8	1.00	1.00
Done	148	0.58 (0.28–1.19)	1.90 (0.85–4.26)
Xpert			
Not Done	79	1.00	1.00
Done	77	3.26 (2.35–4.53)	4.62 (3.18–6.71)

*PTB* Pulmonary tuberculosis

*Xpert* Xpert MTB/RIF

*MTB* Mycobacteria of tuberculosis

Delay of RR-TB detection: days from the first visit to a TB hospital to when rifampicin-resistance was reported

^a^Adjusted for the other factors shown in the multivariate model

## Discussion

To our knowledge, this is the first real-world, population-based study that evaluates the effect of Xpert on PTB and RIF-resistance detection at a provincial level in China. The majority of research on Xpert in China has been confined to laboratories and hospitals [23–26], focusing on the accuracy of Xpert compared to sputum smear microscopy, culture or other molecular methods. Few have studied the performance of Xpert in a routine setting. Our study indicates that within a routine setting in Shanghai, China, Xpert accounted for 11 and 9% increase in bacteriological confirmation and DST coverage. It also increased RR-TB detection by 1% and significantly reduced delay of detection (*p*-value < 0.001).

Our study found a significant increase (11%) in bacteriological confirmation when Xpert was offered to all presumptive patients. This was similar to studies in other countries and regions [12, 27–29], although the proportion varied, ranging from 5% in Zimbabwe [29] to 59% in Brazil [27]. We also noted the relatively low sensitivity of Xpert in this study (88% for smear-positive, culture-positive patients; 64% for smear-negative, culture-positive patients) compared to the findings in a Cochrane review (98% for smear-positive, culture-positive patients; 67% for smear-negative, culture-positive patients) [30]. This difference may be a result of the real-world context compared to clinical trials that control for many variables. The WHO has recommended using Xpert rather than smear microscopy and culture as the initial diagnostic test in all adults suspected of having TB if resources allow [4]. Considering that in our study Xpert missed 23% smear microscopy or culture positive patients, further analysis is needed to identify and eliminate factors associated with the heterogeneity and improve the performance of Xpert before any modification to the diagnosis cascade should be adopted.

We also observed an increase in DST coverage and RR-TB detection in our study due to the use of Xpert, which was similar to other studies [12, 31]. As the GLI and the WHO [3, 22] suggested, the project planned to offer new patients who had a RIF-resistant result from Xpert, a second one to reduce the rate of false positivity, but in our study only 16 (31%) finished the second test. Nevertheless, all the 16 patients were verified as RIF-resistant. Xpert has a sensitivity of 95% and specificity of 98% compared to culture-based DST [3]. Moreover, several studies have found that part of the false positivity was attributed to low-level RIF-resistance that could not be found by culture-based DST [3, 10, 11]. Our results may indicate a high-level reliability of RIF-resistance results from Xpert in the current setting, but further evidence is needed.

In our study, the delay of RR-TB detection was dramatically reduced from 62 to 9 days after implementing Xpert. Findings in other countries [14, 32–34] have suggested that Xpert substantially decreased the delay of RR-TB treatment initiation. Our study did not include data on delay from RR-TB detection to treatment initiation. However, RR-TB detection accounted for 23% of total RR-TB treatment delay in Shanghai [16], therefore, Xpert played an important role in the improvement of RR-TB detection and transmission control. Theoretically, the laboratory procedure of Xpert takes only 2 h [4, 13]. However, when implemented in the real world, time to detection might be prolonged by health service providers, logistics and patients [35]. Although there was a significant median time reduction for RR-TB detection, the fact that it still took 9 days highlights the need to identify and address the barriers in the procedure of RR-TB detection to take full advantage of Xpert technology and reduce even further the time to RR-TB diagnosis.

Universal DST is one component of the End TB strategy. The WHO suggested that DST coverage reach 100 and 90% of TB patients be tested using a WHO-recommended rapid diagnostic (WRD) at the time of diagnosis by 2025 [2]. According to TB report of 2018 [1], the proportion of bacteriologically confirmed cases among PTB patients in China was 32%, while the global average was 56%. In addition, due to the low coverage of DST (12% for new cases and 69% for previously treated cases), there is a large gap between estimated and notified MDR/RR-TB cases (58,000 vs. 13,069). Until now, there is no national policy on the usage of a WRD as the initial diagnostic test for all people presumed to have TB.

Our findings provide critical insights about using Xpert in a routine setting in China. However, TB incidence and TB control resources vary significantly throughout the country. It is notable that the experience in Shanghai should be tailored to fit for other regions in the country accordingly.

There are some limitations in our study. Due to the observational study design, the implementation of Xpert was not controlled, 45% patients did not undergo the test. This might underestimate the effect of Xpert on the detection of PTB and RR-TB. Factors associated with low Xpert implementation should be identified to strengthen the scaling-up in the next phase. Our Xpert project was funded by local government and free for patients. Whether this is sustainable still needs to be determined. Previous studies have indicated that cost-effectiveness varied in different settings and algorithms, e.g. initial use for all presumptive TB cases or use only in sputum smear microscopy negative cases [36–39]. Finally, the HIV infection status was not available. However, Shanghai is a low HIV endemic area in China with a reported incidence of 11.8/100000 population in 2017, therefore, we think it would not bias our findings.

## Conclusion

This study provided useful evidence regarding the potential use of Xpert in a high-burden country to increase PTB and RR-TB detection, as well as decrease RR-TB detection delay. It might be helpful for regions with similar settings when considering integrating this technique into routine PTB and RR-TB case-finding algorithms. Operational level problems should be identified and eliminated to fully utilize the benefit of rapid diagnosis by Xpert.

## Data Availability

The data sets analysed during the current study are available from the corresponding author on reasonable request.

## Abbreviations

CDC: Center for Disease Control and Prevention
CI: Confidence intervals
DST: Drug susceptibility test
GLI: Global Laboratory Initiative
IGRA: Interferon-gamma release assay
IQR: Interquartile range
MDR/RR-TB: Multidrug-resistant/rifampicin-resistant TB
MTB: *Mycobacterium tuberculosis*
NTM: Nontuberculous mycobacteria
PPD: Purified protein derivative
PTB: Pulmonary TB
RIF: Rifampicin
RR: Risk ratio
RR-TB: Rifampicin-resistant TB
TB: Tuberculosis
WRD: WHO-recommended rapid diagnostic
Xpert: Xpert MTB/RIF
